# Virtual Reality Respiratory Biofeedback in an Outpatient Pediatric Pain Rehabilitation Program: Mixed Methods Pilot Study

**DOI:** 10.2196/66352

**Published:** 2025-04-14

**Authors:** Kristin Recker, Julia Silliman, Karolina Gifford, Parth Patel, Lisgelia Santana, Aimee K Hildenbrand, Shreela Palit, Rachel Wasserman

**Affiliations:** 1 College of Medicine, University of Central Florida, Orlando, FL, United States; 2 Department of Graduate Medical Education, Nemours Children's Health System, Orlando, FL, United States; 3 Department of Anesthesiology, Nemours Children's Health, Orlando, FL, United States; 4 Department of Pediatrics, Sidney Kimmel Medical College, Thomas Jefferson University, Philadelphia, PA, United States; 5 Center for Healthcare Delivery Science, Nemours Children's Health, Jacksonville, FL, United States

**Keywords:** chronic pain, adolescents, feasibility, respiratory biofeedback, virtual reality, VR, applied VR respiratory biofeedback, acceptability

## Abstract

**Background:**

Chronic pain in adolescents is a significant and growing concern, as it can have negative implications on physical and psychosocial development. Management can be complicated by the increasing risks associated with opioid misuse, highlighting the need for effective nonpharmacological interventions. Biofeedback is an empirically supported behavioral intervention for chronic pain that targets the self-regulation of physiological responses. Virtual reality (VR) is a novel delivery method for biofeedback that could serve as an engaging and effective platform for adolescents.

**Objective:**

The goal of this study was to assess the feasibility, acceptability, and preliminary effectiveness of integrating a VR-delivered respiratory biofeedback intervention into an outpatient pediatric pain rehabilitation program (PPRP) for adolescents with chronic pain.

**Methods:**

In this pilot study, we recruited 9 participants from those enrolled in the PPRP at Nemours Children’s Hospital. Participants underwent 2 VR respiratory biofeedback sessions per week over a 4-week period using AppliedVR’s “RelieVRx” program. Feasibility was defined as >60% of eligible patients enrolling with at least 80% of VR sessions completed. Acceptability was assessed via validated acceptability questionnaires, with high acceptability defined as an average acceptability rating score >3 on a 5-point Likert scale. Open-ended responses were analyzed via qualitative analysis. Preliminary effectiveness was assessed with questionnaires measuring the quality of life (Pediatric Quality of Life Inventory [PedsQL]) and level of pain interference in daily activities (Functional Disability Inventory) before and after participation in the pain program. Finally, heart rate (HR) and blood pressure (BP) were measured before and after each VR session.

**Results:**

Of 14 eligible PPRP patients, 9 (64%) enrolled in the VR respiratory biofeedback study, and 7 (77% of study participants) completed at least 80% of biofeedback sessions. Participants reported high acceptability with average session ratings ranging from 3.89 to 4.16 on post-VR program questionnaires. Of 224 open-ended responses, participants reported changes in stress and somatic symptoms (ie, pain distraction and breathing regulation). There was a statistically significant increase in the average physical functioning score of the PedsQL among participants (*P*=.01) from pre- to postparticipation in the overall pain program. The cohort’s average emotional functioning score of the PedsQL also increased, though this change was not statistically significant (*P*=.17). Participants’ Functional Disability Inventory scores significantly decreased from an average of 25.1 to 11 from before to after the pain program (*P*=.002). There were no significant differences between pre- versus post-BP or HR for any session. However, decreased BP and HR were observed across most sessions.

**Conclusions:**

AppliedVR respiratory biofeedback demonstrated initial feasibility, acceptability, and preliminary effectiveness when implemented as part of a PRPP. This study underscores the need for future larger-scale studies analyzing the use of VR biofeedback in adolescent populations with chronic pain.

## Introduction

Chronic pain affects upward of 38% of youths and negatively affects multiple aspects of development, including physical and psychological well-being [1-3]. Patients with chronic pain are often initially treated with pharmacotherapy and nonsteroidal medications including ibuprofen, acetaminophen, and in more severe cases, opioids [4]. Given the risks associated with these medications and the misuse of prescription opioids among children and adolescents in the United States, it is imperative to advance nonpharmacological interventions for pain [5,6]. Currently, nonpharmacological treatment for chronic pain in adolescents often involves a multidisciplinary approach encompassing pain education, psychological interventions, integrative medicine (mind-body techniques), and physical and occupational therapies [7,8]. However, these methods can be enhanced. While the etiology of chronic pain is not fully understood, dysregulation of the autonomic nervous system (ANS) is considered an important factor in maintaining many forms of chronic pain [9-12]. There is evidence that stress and anxiety, which trigger sympathetic nervous system responses, can increase pain perception [12]. For example, studies have shown that elevated sympathetic activity is implicated in altering pain perception and sensitivity [11,13]. Activation of the parasympathetic nervous system can help regulate the ANS, decrease pain perception, and increase overall functioning [14,15].

Biofeedback is among the most effective behavioral interventions for chronic pain; with biofeedback training, individuals learn how to control and self-regulate ANS responses such as respiratory rate or heart rate (HR) [16,17]. Visual or auditory cues are provided regarding physiological states, such as breathing rates or patterns [18]. This feedback increases patients’ awareness of the physiological processes occurring in real time inside their bodies and allows them to adjust these processes in a desired direction [19,20]. There are many types of biofeedback modalities, including respiratory, HR variability, thermal, and neurofeedback. For example, during respiratory biofeedback, changes in breathing rate and pattern are monitored and displayed to the patient (eg, on a computer screen). The patient uses this feedback to match their breathing rate and pattern to a computer program that displays a “relaxed” breathing pattern. When the patient’s breathing is smooth and steady (representing an increase in parasympathetic activation), visual feedback from the computer provides positive cues to reward the patient for establishing a relaxed breathing pattern. If the breathing rate becomes irregular at any point, this is indicated on the monitor, and the patient can try to correct their breathing pattern and return to a relaxed pattern.

Recent research demonstrates that biofeedback is effective for adolescents. They are able to significantly modify their respiration rates and reduce muscle tension through increased physiologic control from biofeedback training sessions [21]. While these results are promising, clinical experience suggests that adolescents may lose interest in traditional biofeedback, if it is not sufficiently engaging [22,23]. As a result, efforts are being made to increase pediatric or adolescent engagement in biofeedback therapies through creative platforms like computer games and virtual reality (VR). For example, one study using video game–based biofeedback in adolescent patients showed good retention across 8 sessions and improvement in anxiety and depressive symptoms [24]. VR has the potential to be even more engaging than a computer-delivered biofeedback program because VR limits outside distractions and can increase one’s ability to focus on what is being presented in the VR headset. The use of VR alone, using only relaxing scenes without a biofeedback component, has demonstrated decreases in patients’ perception of pain in both acute and chronic pain management [25,26]. Additionally, advancements in VR technology and increases in the affordability and portability of VR headsets allow for patients to use headsets in various settings and may reduce barriers to participation in biofeedback programs. A technology start-up company, AppliedVR, has developed a respiratory biofeedback intervention delivered with a VR headset that has demonstrated efficacy among adults with chronic lower back pain [27,28]. It is possible that this product could be engaging and effective for adolescents with chronic pain. However, the AppliedVR biofeedback intervention has not yet been studied in this population.

Thus, this pilot study used mixed methods to address this gap by assessing the feasibility, acceptability, and preliminary effectiveness of the AppliedVR, respiratory biofeedback intervention, delivered as part of an integrated pediatric pain rehabilitation program (PPRP) for adolescents with chronic pain. We hypothesized that the AppliedVR, respiratory biofeedback intervention, would be feasible to deliver as part of an existing PPRP. We also expected that the AppliedVR intervention would be rated, on average, as acceptable by adolescents with chronic pain through both quantitative and qualitative assessments. Additionally, we explored the preliminary effectiveness of the VR intervention, such that we anticipated the participants would show improvements in functioning and quality of life before and after the PPRP and decreases in blood pressure (BP) and HR before and after each VR session.

## Methods

### Participants and Recruitment

We aimed to integrate the VR biofeedback sessions into the PPRP at Nemours Children’s Hospital, Florida. The Nemours PPRP is well-established at the hospital and includes 4 weeks of multidisciplinary chronic pain interventions. As a part of this program, patients typically spend 5 hours per day, 5 days per week, for 4 weeks undergoing physical therapy, occupational therapy, individual psychotherapy (including biofeedback), family therapy, adjustments to pain medicine, and weekly team care conferences. The individual psychotherapy sessions occurred once a week throughout the PPRP. While these sessions did not always focus on biofeedback, it was offered when clinically indicated and based on the patient’s personal interest. The goal of including the VR biofeedback sessions was to offer an enhanced, immersive biofeedback relaxation training that could compliment the PPRP by increasing engagement in learning and allowing for the practice of relaxation skills (ie, diaphragmatic breathing) that are typically taught in the individual psychotherapy sessions. The VR intervention aligned with the goals of the overall PPRP, in that both aimed to reduce chronic pain among adolescents and help introduce coping mechanisms that may improve their overall quality of life.

Given that the primary goal of this pilot study was to assess the continuous outcomes of the feasibility and acceptability of the VR intervention, we aimed to recruit a sample size of 15 participants, which is considered adequate for these objectives [29,30]. Eligibility criteria for this pilot study included (1) age range between 13 and 18 years (lower limit of 13 years because the VR headset manufacturer, Oculus, does not recommend the use of the headset by persons 13 years or younger and upper limit of 18 years because only patients 18 years or younger participate in the PPRP), (2) English fluency in verbal and reading or writing (measures were not validated in other languages), (3) participating in the PPRP at Nemours Children’s Hospital during the data collection time frame, and (4) no history of epilepsy or seizures (Oculus does not recommend persons with these conditions to use their VR headset).

As a part of routine clinical care, the PPRP coordinator sends each family a packet with information about the PPRP prior to their participation. While the study was actively recruiting, the families received a study flyer in their pre-PPRP information packet.

### Ethical Considerations

A research team member met with each family in the first week of their participation in the PPRP. During this meeting, the research team member provided information about the research study, answered questions about the study, and obtained written adolescent assent and parental permission for the adolescent to participate. The data was deidentified. This study was approved by the Nemours Children’s Health Institutional Review Board (1552864).

### The VR Intervention

VR biofeedback sessions were conducted using AppliedVR’s “RelieVRx” (formerly “EaseVRx”) application on a portable Oculus Go VR headset [31]. The RelieVRx application included several activities that aim to improve cognitive behavioral and mindfulness-based pain management skills [31]. The software program includes an immersive VR system that incorporates biopsychosocial pain education, diaphragmatic breathing training, mindfulness exercises, and relaxation-response exercises [31]. A microphone on the bottom of the VR headset detects the user’s breath, which is shown in the VR environment as bubbles or a stream of air. In this way, the user learns to regulate their breathing with cues in the VR environment, such as waves of light that move toward and then away from the viewer in a pattern that mimics a relaxed breathing rate.

Consented participants engaged in up to 8 VR biofeedback sessions that occurred each Tuesday and Friday over the course of the 4-week pain program. Each session included 2 activities from the AppliedVR “RelieVRx” application. For the first 2 weeks of the pain program (sessions 1‐4), the research team members (PP, JS, and KR) instructed participants on the specific activities to complete. These specific RelieVRx sessions were selected by a licensed pain psychologist on the research team, as they provided an introduction to the program, built mastery in diaphragmatic breathing, and provided exposure to mindfulness-based attention training. During the final 2 weeks (sessions 5‐8), participants were instructed to choose the RelieVRx content they engaged with, providing them the opportunity to explore other content or revisit prior activities. Activity 1 was divided into 8 sessions. Session 1 was pain care introduction of 3 minutes, session 2 was paced breathing of 6 minutes, session 3 was building breath of 6 minutes, session 4 was deep relaxation of 5 minutes, and sessions 5 to 8 were patient’s choice. Activity 2 began with session 1 breath of hope of 7 minutes, session 2 focus game 1 of 2 minutes, session 3 focus game 2 of 3 minutes, session 4 focus game 3 of 3 minutes, and sessions 5 to 8 patient’s choice.

### Outcome Measures

**Figure 1.**
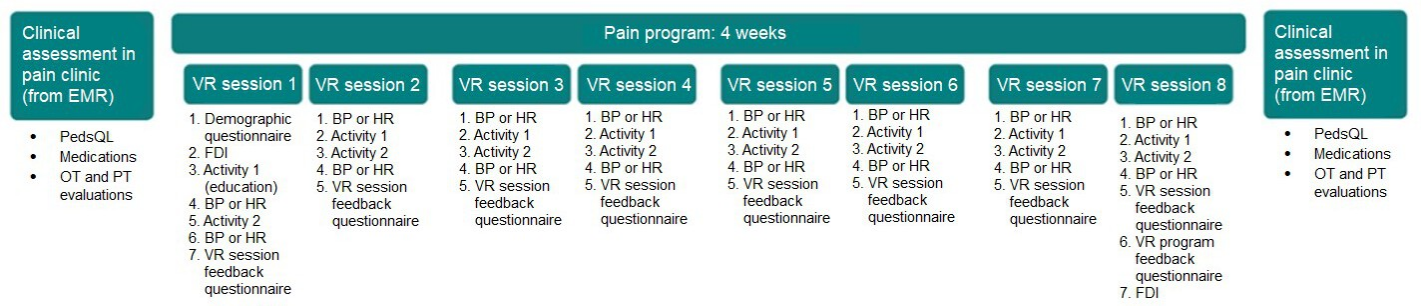
Schedule for outcome measures. BP: blood pressure; EMR: electronic medical record; FDI: Functional Disability Inventory; HR: heart rate; OT: occupational therapy; PedsQL: Pediatric Quality of Life Inventory; PT: physical therapy; VR: virtual reality.

Prior to the PPRP, all patients underwent a clinical assessment as a part of standard clinical practice, and results from the assessment are included in the patient’s electronic medical record (EMR). Patients underwent the same assessment after completing the PPRP. For the purposes of this study, research team members extracted results from the pre- and post-PPRP clinical assessments from each participant’s EMR. Additionally, as a part of the research study, participants were asked to complete questionnaires before the first VR session, after each VR session, and after the final VR session (Figure 1).

Demographic information was assessed via a self-report questionnaire prior to the first VR session. Feasibility was assessed via recruitment and attendance records. We operationalized the feasibility of the VR intervention as >60% of eligible patients enrolling with at least 80% of VR biofeedback sessions completed.

Acceptability was assessed via an acceptability questionnaire designed for this study by a team of clinical psychologists with expertise in measure development. The items were adapted from a validated questionnaire (ie, Acceptability of Intervention Measure) that is commonly used to assess the acceptability of an intervention [32]. The session feedback questionnaire included 5 questions with Likert scale response options (modeled after the Acceptability of Intervention Measure questionnaire) and 3 open-ended questions (developed by the research team). Participants filled out this acceptability questionnaire after completing each VR session. An additional acceptability questionnaire was designed to assess patients’ satisfaction with the VR biofeedback program as a whole. The overall program acceptability questionnaire included a similar structure (5-point Likert scale response items and 5 open-ended questions). We used the same 5-point Likert scale response (1=completely disagree to 5=completely agree) for both acceptability questionnaires. Negatively worded questions were reverse-coded, such that higher ratings indicated higher levels of acceptability. High acceptability was defined as an average acceptability rating score >3 (to capture more positive than “neutral” or negative responses). Open-ended responses were analyzed via qualitative analysis, as described below.

The preliminary effectiveness of the PPRP was assessed via 2 self-reported, validated questionnaires that were completed prior to and after the PPRP. Impairment in daily activities was assessed via the Functional Disability Inventory (FDI) [33,34]. The FDI is a self-report measure of how much pain interferes with day-to-day functioning for adolescents. Adolescents completed the self-report version of this measure prior to their first VR session and immediately after their last VR session. The FDI has excellent psychometric properties [33,34]. Quality of life was assessed with the Pediatric Quality of Life Inventory (PedsQL) Core Generic Scale [35]. The PedsQL is a well-validated and widely used 23-item measure of general quality of life, with separate forms for parental proxy report and a self-report form for youths in the 5‐7, 8‐12, and 13‐18 year age ranges. The PedsQL yields a total score and reliable subscale scores for the child’s physical, emotional, social, and school functioning. The PedsQL was delivered as a part of the standard pre- and postpain program clinical assessment. We obtained total PedsQL scores from the participant’s EMR.

The preliminary effectiveness of the VR sessions was assessed via health outcomes. HR and BP were assessed via a Dinamap Carescape vital signs monitor. Research team members who were second-year medical students conducted each VR session. These team members were trained by a medical assistant on how to use the vitals machine and took noninvasive BP and HR readings using an upper arm cuff placement. These readings were assessed before and after each VR session.

### Statistical Analysis

Demographic and clinical characteristics were summarized using descriptive statistics (mean, SD, and frequencies). Recruitment rate and acceptability questionnaires were analyzed using descriptive statistics. Preliminary effectiveness of VR biofeedback sessions was assessed using paired 2-tailed *t* tests evaluating changes in FDI and PedsQL scores from before and after the pain program as well as BP and HR change scores from each session. Given the main aims of the study were focused on acceptability and feasibility, we accepted any missing data as missing and analyzed means based on the data that were complete. Because surveys were completed with the research coordinator present and checked for completion, we only had 1 participant leave a single item blank on one of the postsession surveys. We dropped this missing item and averaged the remaining responses.

### Qualitative Analysis

We conducted a thematic analysis to analyze open-ended responses on the acceptability questionnaire via the 6-phase approach to the thematic analysis described by Clarke and Braun [36]. First, we compiled all responses to each question into a Microsoft Excel sheet, collapsing across assessment time points. Second, 2 coders (JS and KR) reviewed all qualitative responses and coded each response to create initial codes. Coders were medical students who conducted the VR sessions with study participants. Because the qualitative data were limited due to the small sample size and open-ended questions on the questionnaire were typically short, 1-sentence responses, coding was not divided up but rather all of those involved reviewed all of the data. Although the Clarke and Braun [36] 6-phase approach does not require interrater reliability metrics, having all reviewers review all the data rather than dividing it enhanced the reliability of our coding procedure.

Third, themes were created by identifying broad areas of overlap among codes. Individual codes were collapsed into larger themes by identifying unifying features between them. For instance, codes associated with positive words and phrases about the VR sessions such as “relaxed or calming” were grouped into a larger theme of perceived effects on stress. Fourth, themes were quality-checked and reassessed again. Themes were checked against the extracted data to ensure that they worked in relation to the data. Occasionally, codes were relocated under a different theme, and some themes were collapsed into a broader, more coherent theme. Fifth, themes were named to encompass the overall essence of the individual codes that were grouped into them. Some themes were divided into subthemes. For example, our theme of perceived effects on stress was further split into subthemes of increased stress or decreased stress.

Finally, themes were organized into a flowchart to visualize subthemes with attached example excerpts from questionnaires. The themes and codes were reviewed and revised by the coding team and another research team member who has experience in thematic analysis (RW). Themes were summarized by one of the coders (JS) and distributed to coauthors for review.

## Results

### Feasibility

Over the course of a 12-month period (May 2021 to April 2022), 17 patients enrolled in PPRP at Nemours Children’s Hospital. Of those, 3 were ineligible for the VR study due to age, and 5 declined due to scheduling conflicts or disinterest. Of the 14 eligible patients, 9 consented to the VR respiratory biofeedback study, resulting in a 64% recruitment rate. Participants were between the ages of 13 and 18 years, predominantly female (89%), and identified as Hispanic Latino (22%), non-Hispanic Black or African American (33%), and non-Hispanic White (33%).

In total, 2 participants withdrew from the study (1 after 3 sessions and 1 after 1 session) due to scheduling conflicts or disinterest. Thus, 77% (7/9) of the participants completed at least 80% (7/8) of the biofeedback sessions.

### Acceptability—Quantitative Results

Average acceptability was examined for each session and for the overall VR program. Average session-specific ratings ranged from 3.89 to 4.16, indicating neutral to positive responses (Table 1). For the VR program as a whole, average acceptability ratings ranged from 4.14 to 4.43 (indicating positive to very positive responses).

**Table 1.** Average (mean) acceptability ratings for sessions 1‐8^a^.

Questions	Session 1, mean (SD)	Session 2, mean (SD)	Session 3, mean (SD)	Session 4, mean (SD)	Session 5, mean (SD)	Session 6, mean (SD)	Session 7, mean (SD)	Session 8, mean (SD)
1. The session was too long^b^.	3.56 (1.24)	4.13 (0.83)	4.25 (0.71)	3.71 (1.11)	3.86 (0.90)	3.86 (0.90)	4.14 (0.69)	4.20 (0.45)
2. The session was too short^b^.	3.67 (0.87)	3.75 (1.16)	3.75 (0.89)	3.86 (0.90)	3.86 (0.90)	3.86 (0.90)	4.00 (0.82)	4.00 (0.71)
3. The virtual reality made me feel dizzy or light-headed^b^.	4.13 (1.46)	3.88 (1.13)	4.00 (1.07)	3.86 (1.07)	4.00 (0.82)	3.71 (1.25)	4.00 (1.00)	4.20 (0.45)
4. The session was easy to follow.	4.56 (0.53)	4.12 (0.64)	4.13 (0.83)	4.14 (0.69)	4.14 (0.38)	3.86 (1.07)	4.29 (0.49)	4.00 (0.71)
5. Overall, I liked this session.	4.33 (0.50)	4.38 (0.52)	4.25 (0.71)	4.14 (0.69)	4.57 (0.53)	4.14 (0.69)	4.29 (0.49)	4.40 (0.55)
Average (items 1-5) session acceptability	4.05 (0.43)	4.05 (0.24)	4.08 (0.21)	3.94 (0.19)	4.09 (0.30)	3.89 (0.16)	4.14 (0.14)	4.16 (0.17)

a This table includes responses to a 5-point Likert scale questionnaire (1=completely disagree, 2=somewhat disagree, 3=neither agree nor disagree, 4=somewhat agree, and 5=completely agree.

b The results of questions 1-3 were reverse-coded for consistency, in that higher scores indicate greater acceptability.

### Acceptability—Qualitative Results

**Figure 2.**
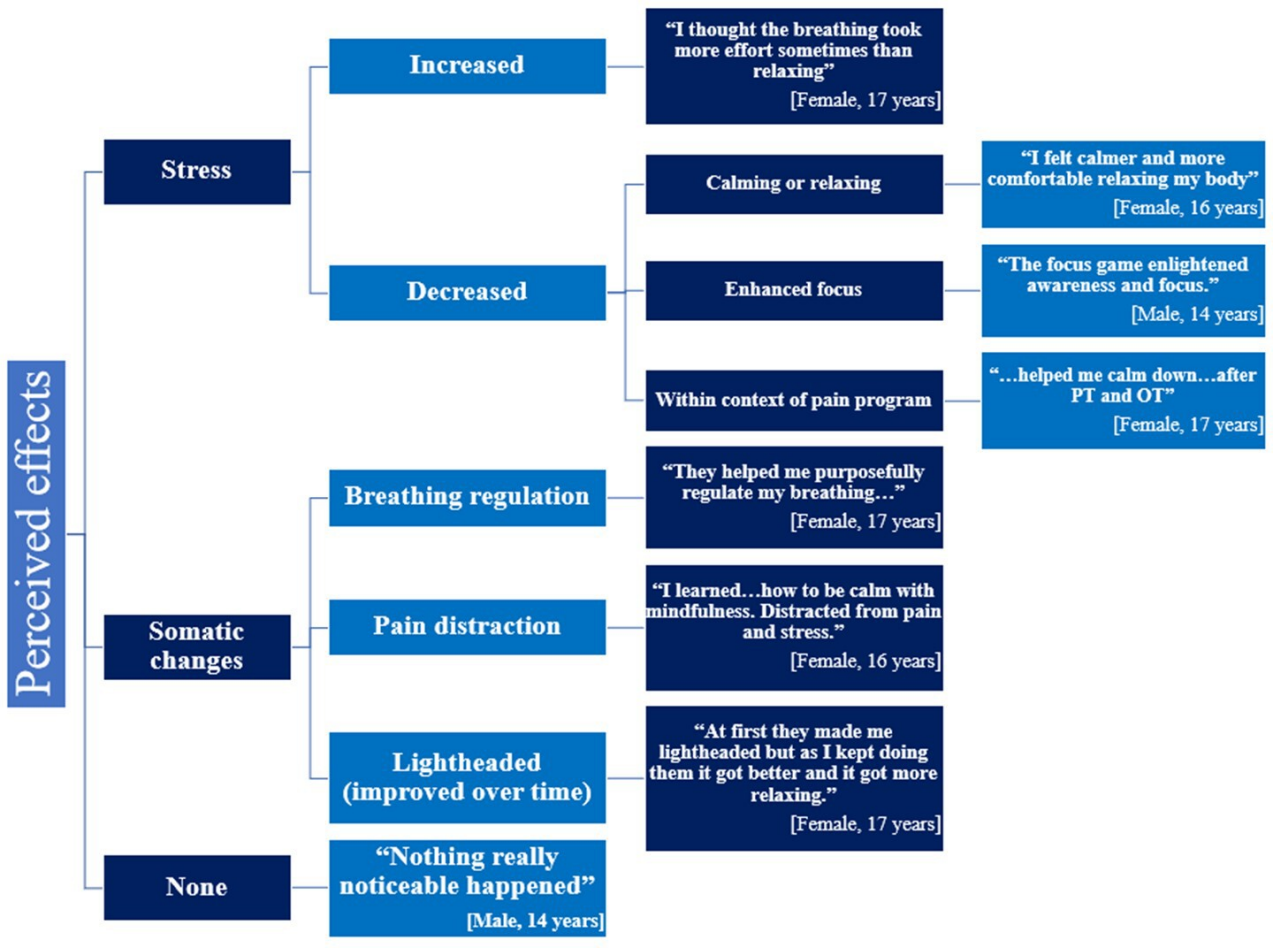
Perceived effects of web-based subcategories. OT: occupational therapy; PT: physical therapy.

Of a total of 224 responses to open-ended questions (provided by 9 participants), themes included (1) perceived effects of VR (eg, stress, no perceived effect, and somatic changes) and (2) VR feedback (eg, general vs specific; Figure 2).

In general, most participants described the VR sessions favorably, reporting that it was calming and relaxing and distracted them from their pain. Additionally, participants endorsed that they had enhanced awareness and focus after the completion of each individual session, and they enjoyed being able to regulate their breathing in a way that interacted with the VR environment. One person reported feeling lightheaded or dizzy during the first couple of VR sessions, though, by the end of the program, she stated that she acclimated to the VR and ultimately enjoyed the VR sessions.

Participants also provided some specific feedback on the sessions (Textbox 1). Participants mainly enjoyed the digital nature of the VR sessions and how the surrounding environment could be changed in relation to their breathing patterns. Participants additionally expressed positive comments about the autonomy to choose their own games and breathing exercises in the last 4 individual sessions, and they liked that the sessions were typically at a relaxing pace (Textbox 1).

Textbox 1. **Sample responses across participants regarding specific feedback subthemes.**

**Length of session and breathing intervals**

- “... make it just a little shorter” [Female, 17 years].
- “in paced breathing the gaps between inhaling seemed a little too long” [Male, 14 years].

**Clarity of instructions**

- “simple, easy to follow” [Male, 14 years].
- “I disliked that the first one did not say what to do, so I assumed to breathe with the tree” [Female, 16 years].

**Amount of interaction**

- “[I liked] the blowing in and out of your breath to move the circular stone” [Female, 13 years].
- “I liked how the breathing ‘class’ synced with the tree and how the greenery of the plants were all around me” [Female, 16 years].

**Voiceover language**

- “I didn’t enjoy the words talking about my energy because I believe Jesus is how you grow and relax” [Female, 17 years].
- “Everything was nice except for the repetitive phrases in the first part” [Female, 16 years].

**Virtual reality scenery and music**

- “I like the scenery and calming music. I liked how it changed between day and night” [Female, 16 years].
- “[I liked] the music and graphics” [Female, 17 years].

**Autonomy of choice and pace**

- “I liked that the first one was kind of on your own and you could go at your own pace” [Female, 17 years].
- “[I liked] the choice of what I could do” [Female, 15 years].

### Preliminary Effectiveness

Paired 2-tailed *t* tests were conducted to examine changes in quality of life (PedsQL) and pain interference (FDI) from baseline to postpain program. PedsQL scores were calculated in terms of both physical and emotional functioning. There was a statistically significant increase in the average physical functioning score among participants (*t*_6_=−3.752; *P*=.01; 95% CI −37.585 to −7.915; effect size [Hedges correction]=17.139) from pre- to postparticipation in the overall program. The cohort’s average emotional functioning score also increased, though this change was not statistically significant (*t*_6_=−1.534; *P*=.18; 95% CI −35.214 to 8.071; effect size [Hedges correction]=25.003; Multimedia Appendix 1). Participant FDI scores significantly decreased from an average of 25.1 to 11 from before to after the pain program (*t*_6_=5.394; *P*=.002; Multimedia Appendix 2).

Paired sample 2-tailed *t* tests assessed differences in systolic BP, diastolic BP, and HR before and after each VR session. We used the Bonferroni correction method to adjust the threshold for significance due to multiple tests (*P≤*.002) [37]. There were no significant differences between pre- versus postsystolic BP, diastolic BP, and HR for any session. However, a signal toward decreased BP was observed across most sessions (see Table 2 for general trends in pre- vs postsession BP and HR). All sessions also resulted in decreased HR, though these changes were not statistically significant.

**Table 2.** Paired 2-tailed *t* test results of the average difference in systolic and diastolic blood pressures (in mm Hg) and heart rate (in bpm) before and after virtual reality (VR) session^a^.

		Pre-VR	Post-VR	*t* test (*df*)	*P* value (2-tailed)
**Session 1**					
	Systolic	112.5	113.4	−0.214 (7)	.84
	Diastolic	65.4	66.1	−0.453 (7)	.66
	Heart rate	88	86	1.239 (7)	.25
**Session 2**					
	Systolic	120.0	114.1	2.056 (6)	.08
	Diastolic	64.7	62.4	2.359 (6)	.05
	Heart rate	90	87	1.094 (6)	.31
**Session 3**					
	Systolic	116.6	112.6	2.828 (7)	.02
	Diastolic	63.1	62.3	0.519 (7)	.62
	Heart rate	88	85	1.449 (7)	.19
**Session 4**					
	Systolic	114.0	113.4	0.272 (6)	.79
	Diastolic	64.6	62.7	0.692 (6)	.51
	Heart rate	88	86	0.721 (6)	.49
**Session 5**					
	Systolic	116.3	117.9	−0.675 (6)	.52
	Diastolic	64.7	63.6	0.481 (6)	.65
	Heart rate	92	88	1.391 (6)	.21
**Session 6**					
	Systolic	119.7	113.1	2.374 (6)	.05
	Diastolic	69.7	67.1	1.140 (6)	.29
	Heart rate	90	86	1.097 (6)	.32
**Session 7**					
	Systolic	119.4	115.4	1.362 (6)	.22
	Diastolic	65.9	64.9	0.544 (6)	.61
	Heart rate	90	90	0.137 (6)	.89
**Session 8**					
	Systolic	112.6	109.6	2.070 (4)	.11
	Diastolic	67.0	60.8	1.583 (4)	.19
	Heart rate	88	82	1.881 (4)	.13

a The threshold for significance was adjusted for multiple testing using the Bonferroni correction method.

## Discussion

### Principal Findings

AppliedVR respiratory biofeedback demonstrated initial feasibility, acceptability, and preliminary effectiveness when integrated into a PPRP. Additionally, open-ended, qualitative feedback on the overall VR program was positive. While not powered to detect changes over time, we did see changes in the expected direction in health outcomes (BP and HR) from pre- to post-VR sessions. The signal of generally decreased BP and HR after VR session is promising and should be further investigated in a large sample with greater power to detect changes.

The PPRP successfully improved reported functioning on 2 different self-report measures (the FDI and the PedsQL). This indicates that adding the VR sessions did not interfere with positive improvements, generally seen in the larger PPRP. Overall, this pilot study demonstrated that a larger implementation or effectiveness trial to assess the impact of integrating a VR-delivered relaxation and mindfulness training program into a PPRP is feasible and would likely be acceptable to adolescents. Our results further suggest that VR platforms may serve as an important tool in keeping adolescents engaged in biofeedback training; however, allowing for customization and personalization within these platforms among users may allow for even higher acceptability and user satisfaction.

### Comparison With Prior Work

Since the completion of this study, 3 other published pilot studies of VR-delivered biofeedback programs have also demonstrated the feasibility and acceptability of using this technology with adolescents in the following settings: inpatient during the perioperative period [38], inpatient for those undergoing port catheter needle insertion [39], and outpatient or home setting for migraines [40]. Each of these 3 studies use slightly different technology and software for the VR and biofeedback components and did not use the same technology as was used in this study. Similar to our findings, the acceptability ratings in these 3 studies were high, with participants reporting that the VR environment was engaging and relaxing [38-40]. One study compared VR with biofeedback to a tablet (iPad)-delivered augmented reality with biofeedback intervention. Interestingly, adolescents reported that both interventions were acceptable but reported a preference for the VR and biofeedback intervention [40]. One unique aspect of this study is that we examined the feasibility of including the VR-delivered intervention as a part of an integrated PPRP. Prior work has examined using VR as an engaging tool in the pediatric rehabilitation setting, but the intervention was focused on increasing movement and range of motion rather than teaching biofeedback and relaxation techniques [41].

### Limitations

The main limitation of this prospective pilot study is the small sample size, which limits generalizability and our ability to examine effectiveness. While our initial sample size goal was 15, reductions in clinical volume correlating to the COVID-19 pandemic restricted participation and recruitment. Given the small sample size, the results of this study may not be applicable to the broader adolescent population. Another limitation of our study is the lack of diversity among student participants with the sample being predominantly female. However, this demographic composition aligns with existing research, which indicates that chronic pain treatment is more prevalent among women and non-Hispanic White people in the adult population, suggesting that our participant demographics are representative of this group [42].

Additionally, because the VR biofeedback intervention in this study was integrated into a PPRP, we were unable to assess the independent effect of VR biofeedback on improvements in adolescent-reported physical or emotional functioning. Furthermore, this study did not include a control group, which will be important in future research to more clearly demonstrate efficacy. In addition, the VR sessions were conducted by medical students as a part of this research study. Thus, these methods may not transfer to a real-world setting, and to assess feasibility for use in an existing pain program, future studies likely will need pain program interventionists to be using the VR program in their sessions rather than having the study team conduct separate sessions. Finally, the AppliedVR program was initially developed for the adult population and has not yet been validated for use with adolescents; thus, while acceptable to adolescents, future research is needed to demonstrate efficacy and effectiveness in this population.

### Conclusions

This prospective pilot study is novel in its use of VR in a PPRP for pediatric chronic pain management and provides a foundation for future larger-scale studies of VR biofeedback. Research into nonpharmacological adjuncts is essential to identify acceptable, feasible, and effective alternatives to chronic pain management. Innovative technologies such as video games, VR, and augmented reality may provide a more immersive experience for biofeedback sessions, but given their novelty, require testing to determine whether it is acceptable and feasible to patients and families. This study provides initial support for the acceptability and feasibility for a VR-based biofeedback in the management of pediatric chronic pain within the context of a multidisciplinary treatment program. More research is needed with larger, more diverse groups of adolescents to further determine the usefulness of such a program and perhaps identify any areas for adaptation and improvement.

## Supplementary material

10.2196/66352 Multimedia Appendix 1 PedsQL before and after the PPRP. PedsQL: Pediatric Quality of Life Inventory; PPRP: pediatric pain rehabilitation program.

10.2196/66352 Multimedia Appendix 2 Average FDI scores before and after the PPRP. FDI: Functional Disability Inventory; PPRP: pediatric pain rehabilitation program.

## Abbreviations

ANS: autonomic nervous system
BP: blood pressure
EMR: electronic medical record
FDI: Functional Disability Inventory
HR: heart rate
PedsQL: Pediatric Quality of Life Inventory
PPRP: pediatric pain rehabilitation program
VR: virtual reality

## References

[1] Eccleston C, Crombez G, Scotford A, Clinch J, Connell H (2004). Adolescent chronic pain: patterns and predictors of emotional distress in adolescents with chronic pain and their parents. Pain.

[2] Hoftun GB, Romundstad PR, Rygg M (2012). Factors associated with adolescent chronic non-specific pain, chronic multisite pain, and chronic pain with high disability: the Young-HUNT Study 2008. J Pain.

[3] King S, Chambers CT, Huguet A (2011). The epidemiology of chronic pain in children and adolescents revisited: a systematic review. Pain.

[4] Poddighe D, Brambilla I, Licari A, Marseglia GL (2019). Ibuprofen for pain control in children: new value for an old molecule. Pediatr Emerg Care.

[5] Hudgins JD, Porter JJ, Monuteaux MC, Bourgeois FT (2019). Prescription opioid use and misuse among adolescents and young adults in the United States: a national survey study. PLoS Med.

[6] Mboma O, Wirth S, Aydin M (2021). The risk of nonsteroidal anti-inflammatory drugs in pediatric medicine: listen carefully to children with pain. Children (Basel).

[7] Harrison LE, Pate JW, Richardson PA, Ickmans K, Wicksell RK, Simons LE (2019). Best-evidence for the rehabilitation of chronic pain part 1: pediatric pain. J Clin Med.

[8] Friedrichsdorf SJ, Giordano J, Desai Dakoji K, Warmuth A, Daughtry C, Schulz CA (2016). Chronic pain in children and adolescents: diagnosis and treatment of primary pain disorders in head, abdomen, muscles and joints. Children (Basel).

[9] Cortelli P, Pierangeli G (2003). Chronic pain-autonomic interactions. Neurol Sci.

[10] Clauw DJ (1995). The pathogenesis of chronic pain and fatigue syndromes, with special reference to fibromyalgia. Med Hypotheses.

[11] Gibbs GF, Drummond PD, Finch PM, Phillips JK (2008). Unravelling the pathophysiology of complex regional pain syndrome: focus on sympathetically maintained pain. Clin Exp Pharmacol Physiol.

[12] Campbell TS, Johnson JA, Zernicke KA, Gellman MD (2020). Encyclopedia of Behavioral Medicine.

[13] Passatore M, Roatta S (2006). Influence of sympathetic nervous system on sensorimotor function: whiplash associated disorders (WAD) as a model. Eur J Appl Physiol.

[14] Busch V, Zeman F, Heckel A, Menne F, Ellrich J, Eichhammer P (2013). The effect of transcutaneous vagus nerve stimulation on pain perception—an experimental study. Brain Stimul.

[15] Sedan O, Sprecher E, Yarnitsky D (2005). Vagal stomach afferents inhibit somatic pain perception. Pain.

[16] Schwartz MS, Andrasik F (2017). Biofeedback: A Practitioner’s Guide.

[17] Sielski R, Rief W, Glombiewski JA (2017). Efficacy of biofeedback in chronic back pain: a meta-analysis. Int J Behav Med.

[18] Dissanayake RK, Bertouch JV (2010). Psychosocial interventions as adjunct therapy for patients with rheumatoid arthritis: a systematic review. Int J Rheum Dis.

[19] Frank DL, Khorshid L, Kiffer JF, Moravec CS, McKee MG (2010). Biofeedback in medicine: who, when, why and how?. Ment Health Fam Med.

[20] Hoffman HG, Richards TL, Van Oostrom T (2007). The analgesic effects of opioids and immersive virtual reality distraction: evidence from subjective and functional brain imaging assessments. Anesth Analg.

[21] Fahrenkamp A, Sim L, Roers L, Canny M, Harrison T, Harbeck-Weber C (2020). An innovative and accessible biofeedback intervention for improving self-regulatory skills in pediatric chronic pain: a pilot study. J Altern Complement Med.

[22] Kothgassner OD, Goreis A, Bauda I, Ziegenaus A, Glenk LM, Felnhofer A (2022). Virtual reality biofeedback interventions for treating anxiety : a systematic review, meta-analysis and future perspective. Wien Klin Wochenschr.

[23] Lüddecke R, Felnhofer A (2022). Virtual reality biofeedback in health: a scoping review. Appl Psychophysiol Biofeedback.

[24] Knox M, Lentini J, Cummings T, McGrady A, Whearty K, Sancrant L (2011). Game-based biofeedback for paediatric anxiety and depression. Ment Health Fam Med.

[25] Ahmadpour N, Randall H, Choksi H, Gao A, Vaughan C, Poronnik P (2019). Virtual reality interventions for acute and chronic pain management. Int J Biochem Cell Biol.

[26] Pourmand A, Davis S, Marchak A, Whiteside T, Sikka N (2018). Virtual reality as a clinical tool for pain management. Curr Pain Headache Rep.

[27] Garcia LM, Birckhead BJ, Krishnamurthy P (2022). Three-month follow-up results of a double-blind, randomized placebo-controlled trial of 8-week self-administered at-home behavioral skills-based virtual reality (VR) for chronic low back pain. J Pain.

[28] Darnall BD, Krishnamurthy P, Tsuei J, Minor JD (2020). Self-administered skills-based virtual reality intervention for chronic pain: randomized controlled pilot study. JMIR Form Res.

[29] Pilot study sample size rules of thumb. NCSS.

[30] Machin D, Campbell MJ, Tan SB, Tan SH (2018). Sample Sizes for Clinical, Laboratory and Epidemiology Studies.

[31] Our product. AppliedVR.

[32] Weiner BJ, Lewis CC, Stanick C (2017). Psychometric assessment of three newly developed implementation outcome measures. Implement Sci.

[33] Claar RL, Walker LS (2006). Functional assessment of pediatric pain patients: psychometric properties of the Functional Disability Inventory. Pain.

[34] Walker LS, Greene JW (1991). The Functional Disability Inventory: measuring a neglected dimension of child health status. J Pediatr Psychol.

[35] Varni JW, Seid M, Kurtin PS (2001). PedsQL 4.0: reliability and validity of the Pediatric Quality of Life Inventory version 4.0 generic core scales in healthy and patient populations. Med Care.

[36] Clarke V, Braun V (2017). Thematic analysis. J Posit Psychol.

[37] Armstrong RA (2014). When to use the Bonferroni correction. Ophthalmic Physiol Opt.

[38] Orgil Z, Karthic A, Bell NF (2024). Use of biofeedback-based virtual reality in pediatric perioperative and postoperative settings: observational study. JMIR Perioper Med.

[39] Savaş EH, Semerci R, Sayın A, Dinçer B, Semiz B, Ürey H (2024). A biofeedback based virtual reality game for pediatric population (BioVirtualPed): a feasibility trial. Semin Oncol Nurs.

[40] Connelly M, Boorigie M, McCabe K (2023). Acceptability and tolerability of extended reality relaxation training with and without wearable neurofeedback in pediatric migraine. Children (Basel).

[41] Griffin A, Wilson L, Feinstein AB (2020). Virtual reality in pain rehabilitation for youth with chronic pain: pilot feasibility study. JMIR Rehabil Assist Technol.

[42] (2019). National Health Interview Survey. National Center for Health Statistics.

