# Supplementary figures and images for: Virtual Reality Respiratory Biofeedback in an Outpatient Pediatric Pain Rehabilitation Program: Mixed Methods Pilot Study
Source: JMIR Rehabil Assist Technol. 2025 Apr 14;12:e66352. doi: 10.2196/66352 (PMC12011315; doi:10.2196/66352)

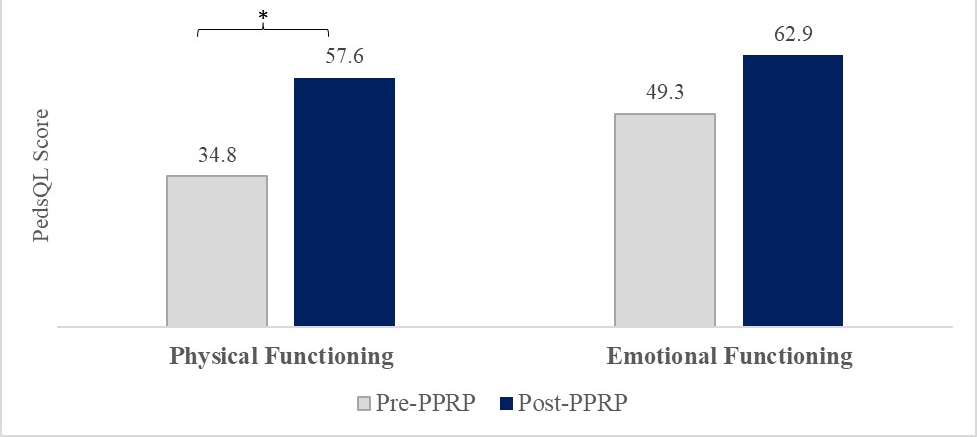

Supplement: Multimedia Appendix 1 [file rehab-v12-e66352-s001.png]

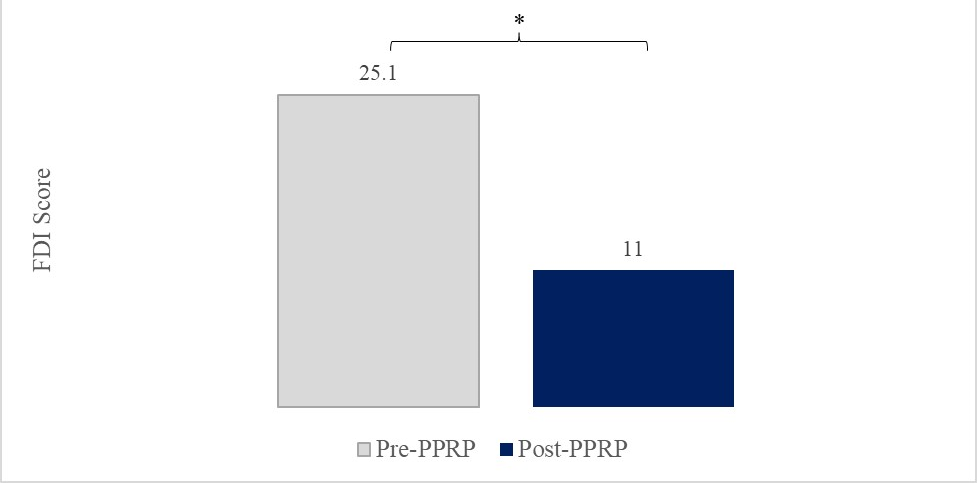

Supplement: Multimedia Appendix 2 [file rehab-v12-e66352-s002.png]
